# Vascular risk factor associations with subjective cognitive decline and mild behavioural impairment

**DOI:** 10.1093/braincomms/fcaf163

**Published:** 2025-04-28

**Authors:** Dylan X Guan, Aditya Aundhakar, Sarah Tomaszewski Farias, Clive Ballard, Byron Creese, Anne Corbett, Ellie Pickering, Pamela Roach, Eric E Smith, Zahinoor Ismail

**Affiliations:** Cumming School of Medicine, University of Calgary, Calgary, Canada, T2N4N1; Hotchkiss Brain Institute, University of Calgary, Calgary, Canada, T2N4N1; Department of Clinical Neurosciences, University of Calgary, Calgary, Canada, T2N4N1; Department of Neurology, Davis School of Medicine, University of California, Sacramento 95817, USA; Clinical and Biomedical Sciences, Faculty of Health and Life Sciences, University of Exeter, Exeter EX44QJ, UK; Department of Psychiatry, College of Health Medicine and Life Sciences, Brunel University London, London UB83PH, UK; Clinical and Biomedical Sciences, Faculty of Health and Life Sciences, University of Exeter, Exeter EX44QJ, UK; Clinical and Biomedical Sciences, Faculty of Health and Life Sciences, University of Exeter, Exeter EX44QJ, UK; Cumming School of Medicine, University of Calgary, Calgary, Canada, T2N4N1; Hotchkiss Brain Institute, University of Calgary, Calgary, Canada, T2N4N1; Department of Family Medicine, University of Calgary, Calgary, Canada, T2N4N1; Department of Community Health Sciences, University of Calgary, Calgary, Canada, T2N4N1; O’Brien Institute for Public Health, University of Calgary, Calgary, Canada, T2N4N1; Cumming School of Medicine, University of Calgary, Calgary, Canada, T2N4N1; Hotchkiss Brain Institute, University of Calgary, Calgary, Canada, T2N4N1; Department of Clinical Neurosciences, University of Calgary, Calgary, Canada, T2N4N1; Department of Community Health Sciences, University of Calgary, Calgary, Canada, T2N4N1; Cumming School of Medicine, University of Calgary, Calgary, Canada, T2N4N1; Hotchkiss Brain Institute, University of Calgary, Calgary, Canada, T2N4N1; Department of Clinical Neurosciences, University of Calgary, Calgary, Canada, T2N4N1; Clinical and Biomedical Sciences, Faculty of Health and Life Sciences, University of Exeter, Exeter EX44QJ, UK; Department of Community Health Sciences, University of Calgary, Calgary, Canada, T2N4N1; O’Brien Institute for Public Health, University of Calgary, Calgary, Canada, T2N4N1; Department of Psychiatry, University of Calgary, Calgary, Canada, T2N4N1; Department of Pathology and Laboratory Medicine, University of Calgary, Calgary, Canada, T2N4N1

**Keywords:** vascular risk factors, diabetes, smoking, subjective cognitive decline, mild behavioural impairment

## Abstract

Subjective cognitive decline and mild behavioural impairment identify older persons more likely to have early Alzheimer’s disease. Vascular co-pathologies may also contribute to new onset and persistent cognitive and behavioural symptoms later in life. We investigated vascular risk factor associations with subjective cognitive decline and mild behavioural impairment. Cross-sectional data for 1285 (81.0% female) participants without mild cognitive impairment or dementia enrolled in the Canadian Platform for Research Online to Investigate Health, Quality of Life, Cognition, Behaviour, Function, and Caregiving in Aging were analyzed. Vascular risk factors included body mass index class, self-reported clinician diagnoses of hypertension, high cholesterol, diabetes, self-reported smoking, and the cumulative number of vascular risk factors. Outcomes were the Everyday Cognition scale and Mild Behavioural Impairment Checklist. Logistic and negative binomial regressions were used to model odds and severity of subjective cognitive decline and mild behavioural impairment as a function of individual or cumulative vascular risk factors. Having three or more vascular risk factors (odds ratio = 1.23, 95% confidence interval [1.04–1.47]), actively smoking (odds ratio = 1.54, 95% confidence interval [1.29–1.82]), being overweight (odds ratio = 1.46, 95% confidence interval [1.22–1.74]), and having diabetes (odds ratio = 1.29, 95% confidence interval [1.09–1.53]) were associated with higher odds of subjective cognitive decline. Having any number of vascular risk factors was dose-dependently associated with higher odds of mild behavioural impairment, as were all five vascular risk factors individually; active smokers (odds ratio = 2.67, 95% confidence interval [2.25–3.18]) and obese persons (odds ratio = 2.29, 95% confidence interval [1.91–2.75]) had over twice the odds of mild behavioural impairment. Vascular risk factors associations with subjective cognitive decline were stronger in participants with mild behavioural impairment. All vascular risk factors were linked to higher Everyday Cognition and Mild Behavioural Impairment Checklist total scores, indicating greater subjective cognitive decline and mild behavioural impairment symptom severity. Overweight body mass index, hypertension, and high cholesterol associations with subjective cognitive decline and mild behavioural impairment were stronger in middle-aged adults than older adults, but diabetes and active smoking had greater effects in older adults. Vascular risk factors are strongly related to experiences of cognitive and behavioural changes in later life, even in the absence of objective cognitive impairment. Furthermore, vascular associations with subjective cognitive decline symptoms may be more pronounced in persons with concomitant behavioural decline. Vascular pathologies may contribute to both cognitive and behavioural markers traditionally linked to Alzheimer’s disease in older persons, prior to mild cognitive impairment and dementia.

## Teaser

Guan *et al*. report that vascular risk factors, including higher body mass index, hypertension, high cholesterol, diabetes, and active smoking, are associated with subjective cognitive decline and mild behavioural impairment, prior to mild cognitive impairment and dementia. They conclude that early mitigation of these factors may reduce future dementia risk.

## Graphical Abstract

**Graphical Abstract:**
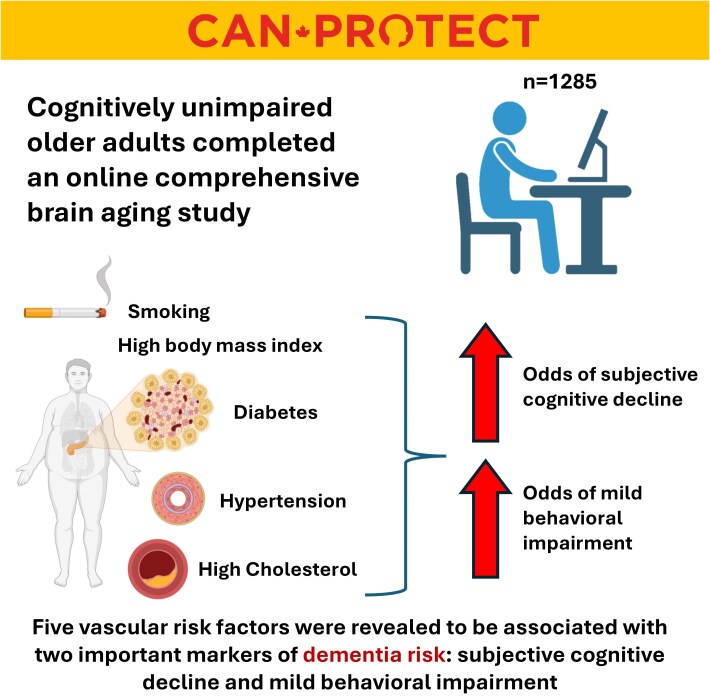

## Introduction

Dementia exacts a profound toll on affected persons and their loved ones and on the healthcare system. Dementia prevalence is expected to surge,^1^ in part due to advancements in modern healthcare that have effectively extended human longevity. Despite these advancements, a definitive cure for dementia remains elusive.^2^ However, dementia risk may be modifiable.^3,4^ Systematic reviews and meta-analyses have identified a range of modifiable risk factors associated with dementia risk, including low educational attainment, hearing loss, social isolation, depression, physical inactivity, body mass index (BMI), hypertension, diabetes, and smoking.^5^ Addressing these risk factors, many of which are also vascular risk factors (VRFs), may hold promise for averting or postponing the onset of dementia.

The concept of mild cognitive impairment (MCI) has long been recognized as an approach to identify a group enriched for prodromal Alzheimer’s disease.^6^ MCI may be preceded by subjective cognitive decline (SCD), which refers to an individual's self-perception of cognitive decline without objective cognitive impairment on standardized neuropsychological tests.^7^ Epidemiological studies show SCD progression rates to MCI and dementia of 27 and 14% over four years, respectively, with SCD preceding dementia by up to 10 years.^8^ Moreover, both biomarker and neuropathological studies indicate that persons with SCD tend to have greater amyloid and tau pathological burden compared to cognitively unimpaired persons without SCD, thus identifying a group enriched for preclinical Alzheimer’s disease.^9-11^

Changes in behaviour are also core features of Alzheimer’s disease, which can even present in advance of dementia, absent cognitive symptoms, or concurrently with subjective (i.e. SCD) or objective (i.e. MCI) cognitive decline. To better describe these pre-dementia neuropsychiatric symptoms (NPSs) and to standardize assessment, the International Society to Advance Alzheimer’s Research and Treatment-Alzheimer’s Association (ISTAART-AA) published diagnostic criteria for mild behavioural impairment (MBI) in 2016.^12^ NPSs meeting these criteria (i.e. later-life emergence representing a change from longstanding patterns of behaviour or personality, and persistence for >6 months) have been shown to predict cognitive decline and incident dementia across several studies,^13-17^ even when compared to NPSs not meeting MBI criteria.^18-21^ When applied to any cognitive category (i.e. normal cognition, SCD, or MCI), MBI acts as an effect modifier, refining the at-risk group to select those who will develop cognitive decline and dementia faster than persons in that category without MBI.^20,22-24^ Beyond dementia risk, MBI is also associated with poorer gait,^25^ impaired hearing,^26^ diabetes,^27^ frailty,^28,29^ loneliness,^30^ sleep disturbance,^31^ and poorer quality of life,^32^ suggesting that MBI itself be an important target for preventive or therapeutic intervention.^33^

Although both SCD and MBI were initially conceptualized as syndromes that identify persons at risk for early-stage Alzheimer’s disease, it is increasingly being recognized that vascular pathology may contribute to, and exacerbate, cognitive and behavioural symptoms in older persons. Indeed, the majority of older persons with dementia also exhibit vascular burden.^34^ Several studies have observed associations between SCD and features of vascular brain pathology, such as white matter hyperintensities,^35-37^ and evidence of vascular links with MBI is also emerging.^38^ These findings indicate that secondary prevention programmes targeting vascular pathology may reduce overall cognitive and behavioural symptom burden. Indeed, individual VRFs and their aggregated burden are strongly associated with imaging markers of cerebral small vessel disease,^39^ and the declining incidence of dementia in several high-income nations has been partly attributed to improved management of VRFs.^40^ It is now well-accepted that several VRFs in midlife are linked to cognitive decline and greater dementia risk, including obesity, hypertension, high cholesterol, diabetes, and smoking, supported both by observational studies and clinical trials, though some of these associations may be attenuated at older ages and disease stages.^5^ Several studies have also shown that the combined burden of several VRFs, often operationalized in a study-specific manner, is also associated with cognitive decline and dementia,^41,42^ implicating interventions that target multiple VRFs concurrently.^43^

However, the relationship between VRFs and the earliest signs of cognitive and behavioural decline, namely SCD and MBI, has not yet been fully explored, especially among community-dwelling older persons. A better understanding of these associations is warranted as SCD and MBI are themselves associated with poorer immediate health outcomes^25-32^ and manifest during early stages of potential disease when participants are more physically and mentally able to follow lifestyle interventions targeting multiple VRFs. Here, we investigate associations between several VRFs, namely obesity, hypertension, high cholesterol, diabetes, and smoking, and the earliest cognitive and behavioural signs of potential neurodegenerative disease, SCD and MBI. We hypothesize that the presence of VRFs is associated with greater odds of, and more severe, SCD or MBI among a cognitively healthy cohort without MCI or dementia.

## Materials and methods

### Study design

The Canadian Platform for Research Online to Investigate Health, Quality of Life, Cognition, Behaviour, Function, and Caregiving in Aging (CAN-PROTECT) is an online longitudinal observational cohort study, focusing on dementia-free community-dwelling individuals aged ≥18 years. Participants were recruited from a variety of sources including local communication channels (media publicity, university press partners, online content); existing study cohorts and trials; advertisements in clinics; strategic targeting of nation-wide seniors’ and Alzheimer's Disease resource centres; and social media platforms. Annually, participants and their study partners are asked to complete assessments of demographics, health, cognition, behaviour, function, lifestyle, and more. Participants are given up to six months from registration to complete all assessments, and several assessments are optional. Informed consent was obtained from all participants as part of the online registration process and in alignment with the Declaration of Helsinki. Ethics approval for the study was obtained from the Conjoint Health Research Ethics Board at the University of Calgary (REB21-1065). A detailed description of the study procedure and existing cohort has been published elsewhere.^44^

### Participants

**Figure 1:**
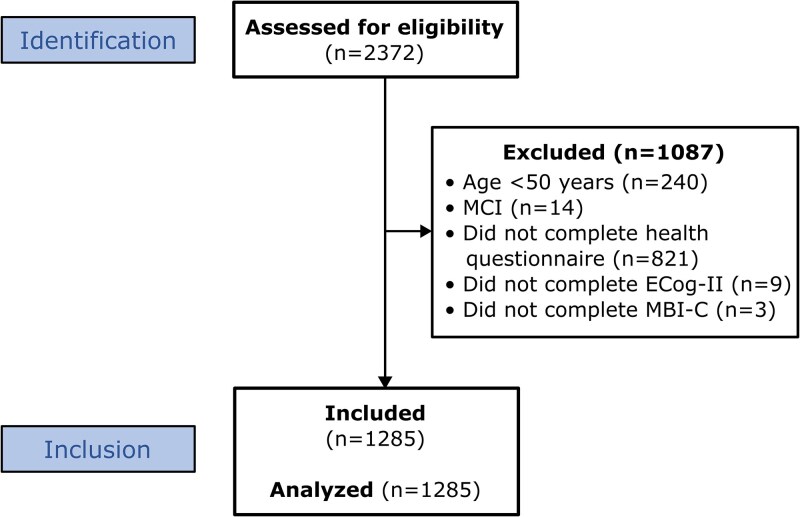
**Participant flow diagram.** Abbreviations: MCI, mild cognitive impairment; ECog-II, Everyday Cognition Scale II; MBI-C, Mild Behavioural Impairment Checklist.

We used baseline data from 1285 participants aged 50 years or older who completed the relevant assessments on demographics, health, lifestyle, cognition, and behaviour. A flow diagram detailing the inclusion/exclusion criteria is shown in Fig. 1. After considering 2372 participants for eligibility, 240 were excluded for being younger than 50 years old, as per the ISTAART MBI criteria,^12^ 14 for having received a clinical diagnosis of MCI, and the remainder for not yet completing the health questionnaire (*n* = 821), revised Everyday Cognition (ECog-II) scale (*n* = 9), or Mild Behavioural Impairment Checklist (MBI-C; *n* = 3) at the time of data abstraction.

### Measures

VRFs included BMI, self-reported clinical diagnoses of hypertension, diabetes, or high cholesterol, and smoking. BMI (kg/m^2^) was derived from self-reported height and weight and then was categorized into underweight (BMI < 18.5), normal (BMI 18.5–25), overweight (BMI 25–30), or obese (BMI ≥ 30).^45^ Smoking was categorized into three levels: never smoked, past smoker, and active smoker. Furthermore, given evidence suggesting that cumulative number of VRFs may also be associated with dementia risk in a dose-dependent manner,^41,42^ we generated a cumulative VRF burden variable specific to this study based on the number of VRFs present (0 VRFs—absent, 1 VRF—low burden, 2 VRFs—moderate burden, ≥ 3 VRFs—high burden).

Cognition was measured using the self-reported ECog-II scale, which evaluates changes in everyday cognition and function along the domains of memory (nine items), language (nine items), visuospatial function (eight items), and executive function (15 items), collapsed across organizational, planning, and divided attention complaints. Participants score each item based on a four-point scale: 0 = no change, 1 = occasionally worse, 2 = consistently a little worse; 3 = consistently much worse relative to a participant’s own baseline.^46^ Participants were classified as having SCD if they scored any ECog-II item 2 or greater (i.e. consistently a little or much worse).^47^ The total ECog-II score was calculated as the sum of all item scores.

The self-reported MBI-C, which has been validated in online settings,^48^ was used to measure MBI symptoms in older persons according to the ISTAART-AA MBI research diagnostic criteria.^12,49^ Participants identify whether symptoms are present, and if so, score them from a scale of 1–3. Symptoms are categorized into five domains: decreased motivation (six items), affective dysregulation (six items), impulse dyscontrol (12 items), social inappropriateness (five items) and abnormal perception or thought content (five items). The sum of item scores within each domain served as the domain scores and the sum of all item scores corresponded to global MBI symptom severity (range = 0–104). Participants who scored 8 or greater on the MBI-C were classified as MBI + .^50^

ISTAART-AA MBI criteria stipulate that psychiatric conditions cannot better account for symptoms.^12^ In legacy datasets with conventional measures of psychiatric and/or neuropsychiatric symptomatology, this MBI criterion was operationalized by excluding participants with a history of psychiatric conditions. As we used the MBI-C as an outcome measure, this step was not required; the MBI-C states explicitly that symptoms emerge *de novo* in later life and represent change from longstanding patterns of behaviour or personality, intrinsically excluding non-MBI NPS.^49^

### Statistical analysis

Demographic, VRFs, and outcome measures were summarized using means, standard deviations (SDs), ranges, and percentages. To compare participants across different levels of VRF burden, we used ANOVA for continuous variables or chi-squared tests for categorical variables. Because several CAN-PROTECT questionnaires are optional, we compared the demographics of participants who were excluded due to incomplete assessments with those included for analysis to identify potential bias.

Because many VRFs correlate with each other with complex causal relationships (e.g. BMI, diabetes, and hypertension are all consequences of the metabolic syndrome),^51^ we built separate models for each of the five VRFs, as well as for cumulative VRF burden. In each model, one of the five VRFs, or cumulative VRF burden, was treated as the exposure variable with SCD status, MBI status, ECog-II score, or MBI-C score as the outcome variable (i.e. four separate outcomes), resulting in a total of 30 models and 40 comparisons (as BMI and smoking status variables each had three levels, and cumulative VRF burden had four levels). Also of interest were also whether associations between VRFs and SCD wdiffered according to MBI status. Therefore, we explored the effect of including VRF×MBI status multiplicative interaction terms into models of SCD status as a function of a VRF as part of a secondary analysis.

We modelled SCD and MBI statuses as a function of VRFs using logistic regression. To model the continuous ECog-II and MBI-C total scores as outcomes, we applied negative binomial regression as the distribution of ECog-II and MBI-C total scores both resembled Poisson distributions (i.e. right-skewed whole numbers) with overdispersion (variance > mean). Both logistic regression and negative binomial regression coefficients were exponentiated to facilitate interpretation; odds ratios (ORs) were reported for logistic regression and exp[b] (i.e. factor change in ECog-II or MBI-C total score in those with a VRF versus those without) was reported for negative binomial regression.

To reduce the risk of Type I error due to multiple comparisons, we adjusted the 40 primary *P*-values of interest using the Benjamini-Hochberg procedure based on false discovery rate (FDR). We refer to FDR-corrected *P*-values as *q*-values, and two-tailed *q*-values that were less than 0.05 were considered statistically significant. Propensity scores with inverse probability treatment weighting (IPTW) were used to balance observed covariates including age, sex, years of education, marital status, and ethnocultural origins across exposure groups. These propensity score weights were used to adjust each regression model accordingly. We verified the performance of IPTW by inspecting the coefficient of variance, effective sample sizes, and standardized mean differences of covariates.

To further understand associations between VRFs, SCD, and MBI, we subsequently conducted two secondary analyses, both exploratory in nature and therefore not included in the correction for multiple comparisons. A subgroup analysis compared middle-aged (<65 years) and older adults (≥65 years), and a domain analysis investigated associations between VRFs and individual ECog-II and MBI-C domains. All analyses were conducted using R version 4.3.0.

## Results

### Sample characteristics

Table 1 presents sample characteristics for the total cohort and stratified by VRF burden (*n* = 1285). The cohort was mostly female (81.0%) and tended to report being married (78.2%), with a mean ± SD age of 64.6 ± 7.4 years and 15.8 ± 4.5 years of education. Participants mostly identified as having North American (47.9%) and/or European (84.2%) ethnocultural origins. Included participants were more likely to have been assigned female sex at birth (81.0% versus 74.1%, *P* < 0.001) and to be married (78.2 versus 73.1%, *P* = 0.008) than those excluded for not completing the relevant assessments. The two groups did not differ in the other demographic variables.

**Table 1:** Participant characteristics stratified by cumulative vascular risk factor burden

Variable	Total	None (0)	Low (1)	Moderate (2)	High (3+)	*P*
*n*	1285	582	399	203	101	
Age (years)	64.6 (7.4), 50–89	63.5 (7.2), 50–89	65 (7.3), 50–87	66.2 (7.6), 50–88	66 (7.5), 50–88	<0.001
Female sex	1041 (81)	497 (85.4)	321 (80.5)	149 (73.4)	74 (73.3)	<0.001
Education (years)	15.8 (4.5), 0–30	16.1 (4.4), 2–30	15.6 (4.9), 1–30	15.4 (4.3), 0–30	15.7 (4), 1–30	0.26
Ethnocultural origins						
European	1082 (84.2)	498 (85.6)	333 (83.5)	171 (84.2)	80 (79.2)	0.41
North American	616 (47.9)	281 (48.3)	177 (44.4)	104 (51.2)	54 (53.5)	0.24
Caribbean	10 (0.8)	5 (0.9)	1 (0.3)	3 (1.5)	1 (1)	0.42
South American	9 (0.7)	3 (0.5)	2 (0.5)	4 (2)	0 (0)	0.11
African	11 (0.9)	4 (0.7)	2 (0.5)	3 (1.5)	2 (2)	0.36
Asian	41 (3.2)	19 (3.3)	12 (3)	6 (3)	4 (4)	0.96
Oceanic	6 (0.5)	4 (0.7)	1 (0.3)	1 (0.5)	0 (0)	0.69
BMI						
Normal (18.5–24.9 kg/m^2^)	547 (42.6)	368 (63.2)	131 (32.8)	41 (20.2)	7 (6.9)	<0.001
Overweight (25–29.9 kg/m^2^)	399 (31.1)	214 (36.8)	129 (32.3)	45 (22.2)	11 (10.9)	
Obese (≥30 kg/m^2^)	339 (26.4)	0 (0)	139 (34.8)	117 (57.6)	83 (82.2)	
Marital status	1005 (78.2)	475 (81.6)	298 (74.7)	164 (80.8)	68 (67.3)	0.002
Hypertension	360 (28)	0 (0)	121 (30.3)	147 (72.4)	92 (91.1)	<0.001
High cholesterol	302 (23.5)	0 (0)	111 (27.8)	107 (52.7)	84 (83.2)	<0.001
Diabetes	87 (6.8)	0 (0)	13 (3.3)	26 (12.8)	48 (47.5)	<0.001
Smoking						<0.001
Never	695 (54.1)	347 (59.6)	211 (52.9)	100 (49.3)	37 (36.6)	
Past	550 (42.8)	235 (40.4)	173 (43.4)	94 (46.3)	48 (47.5)	
Active	40 (3.1)	0 (0)	15 (3.8)	9 (4.4)	16 (15.8)	
History of heart attack	59 (4.6)	15 (2.6)	18 (4.5)	16 (7.9)	10 (9.9)	<0.001
History of stroke	18 (1.4)	4 (0.7)	4 (1.0)	5 (2.5)	5 (5.0)	0.004
SCD status	343 (26.7)	146 (25.1)	109 (27.3)	57 (28.1)	31 (30.7)	0.60
ECog-II Total	11.6 (11.2), 0–99	10.5 (9.9), 0–74	11.8 (11.4), 0–88	13 (13), 0–99	14.3 (12.9), 0–65	0.002
MBI status	312 (24.3)	105 (18)	110 (27.6)	61 (30)	36 (35.6)	<0.001
MBI-C severity	5.2 (7.2), 0–65	4.3 (6.2), 0–44	5 (5.9), 0–39	6.9 (10), 0–65	7.5 (9.3), 0–41	<0.001

*Note*. Ethnocultural origins are not mutually exclusive. All values have been rounded to one decimal place, except for *P*-values which have been rounded to two or three decimal places, as appropriate. Continuous variables are shown in mean (standard deviation), range. Categorical variables are shown in *n* (%). Comparisons between groups were tested using ANOVA for continuous variables and chi-square tests for categorical variables, as appropriate. Abbreviations: BMI, body mass index; SCD, subjective cognitive decline; ECog-II, Everyday Cognition II scale; MBI, mild behavioural impairment; MBI-C, Mild Behavioural Impairment Checklist.

Nearly half (45.3%) of participants reported no VRFs, 31.0% reported having one VRF, 15.8% reported having two VRFs, and the remaining 7.9% reported having three or more VRFs. Based on BMI thresholds, 42.6% of participants had a normal BMI (BMI 18.5–25), 31.1% were overweight (BMI 25–30), and 26.4% were obese (BMI ≥ 30). A clinical diagnosis of hypertension, high cholesterol, and diabetes were reported by 28.0, 23.5, and 6.8% of participants, respectively. More than half of participants had never smoked (54.1%), whereas 42.8% were past smokers, and 3.1% were active smokers.

### Prevalence and severity of SCD and MBI

SCD (defined as at least one consistently worse cognitive complaint) was present in 26.7% of the entire cohort, with an average ECog-II total score of 11.6 ± 11.2. The most prevalent symptoms of SCD were changes in memory (21.2%), followed by language (12.6%), executive function (10.5%), and visuospatial function (3.4%) (Supplementary Table 1). MBI was present in 24.3% of all participants, with an average MBI-C total score of 5.2 ± 7.2. Decreased motivation was the most frequently endorsed MBI domain (23.8%), followed by affective dysregulation (23.7%), impulse dyscontrol (22.8%), abnormal perception or thought content (9.8%), and social inappropriateness (9.0%). Participants with SCD did not always report MBI and vice versa: 61.6% of participants reported neither SCD nor MBI, 14.2% reported SCD in the absence of MBI, 11.8% reported MBI in the absence of SCD, and 12.5% reported both SCD and MBI. Nevertheless, participants with MBI had 4.63 (95% CI [3.52, 6.10], *P* < 0.001) greater odds of SCD, and every 1-point rise in MBI-C total score was associated with a 1.05 (95% CI [1.05, 1.06], *P* < 0.001) times greater ECog-II total score.

### VRF associations with SCD and MBI

Having three or more VRFs were associated with 23% higher odds of having SCD (OR = 1.23, 95% CI [1.04–1.47], *q* = 0.02) relative to persons with no VRFs. In contrast, persons with one or two VRFs did not significantly differ from persons with no VRFs in terms of the odds of having SCD (Table 2). Across the five individual VRFs, three were associated with higher odds of having SCD. The largest effect was observed for active smokers (OR = 1.54, 95% CI [1.29–1.82], *q* < 0.001) and persons classified as overweight according to BMI thresholds (OR = 1.46, 95% CI [1.22–1.74], *q* < 0.001). Diabetes and obesity were also associated with a 29% (OR = 1.29, 95% CI [1.09–1.53], *q* = 0.005) and 26% (OR = 1.26, 95% CI [1.05–1.50], *q* = 0.01) greater odds of SCD, respectively. However, hypertension (OR = 1.14, 95% CI [0.96–1.36], *q* = 0.15), past smoking (OR = 1.13, 95% CI [0.95–1.35], *q* = 0.17), and high cholesterol (OR = 1.09, 95% CI [0.92–1.30], *q* = 0.33) were not associated with the presence of SCD. Subgroup analyses revealed that these associations were generally stronger among middle-aged (<65 years) adults and attenuated or no longer statistically significant in older adults (≥65 years), particularly for overweight BMI and hypertension (Supplementary Table 2).

**Table 2:** Vascular risk factor associations with subjective cognition decline

Outcome Exposure	OR	95% CI	*p/q*
**SCD status**			
Cumulative VRF burden			
Low (1)	1.14	0.96–1.36	0.14/0.15
Moderate (2)	1.17	0.98–1.39	0.09/0.10
High (3+)	1.23	1.04–1.47	0.02/0.02
BMI (ref. normal BMI)			
Overweight	1.46	1.22–1.74	<0.001/<0.001
Obese	1.26	1.05–1.50	0.01/0.01
Hypertension	1.14	0.96–1.36	0.13/0.15
High cholesterol	1.09	0.92–1.30	0.32/0.32
Diabetes	1.29	1.09–1.53	0.003/0.005
Smoking (ref. Never)			
Past smoker	1.13	0.95–1.35	0.16/0.17
Active smoker	1.54	1.29–1.82	<0.001/<0.001

	exp(b)	95% CI	*p/q*
**ECog-II total score**			
Cumulative VRF burden			
Low (1)	1.13	1.05–1.22	<0.001/<0.001
Moderate (2)	1.24	1.15–1.33	<0.001/<0.001
High (3+)	1.41	1.31–1.52	<0.001/<0.001
BMI (ref. normal BMI)			
Overweight	1.22	1.13–1.31	<0.001/<0.001
Obese	1.25	1.17–1.35	<0.001/<0.001
Hypertension	1.15	1.07–1.24	<0.001/<0.001
High cholesterol	1.10	1.02–1.17	0.01/0.01
Diabetes	1.40	1.30–1.51	<0.001/<0.001
Smoking (ref. Never)			
Past smoker	1.00	0.93–1.08	0.93/0.93
Active smoker	1.31	1.22–1.41	<0.001/<0.001

Odds ratios (ORs) were estimated from logistic regression; they indicate the factor change in odds of having SCD between participants with a vascular risk factor relative to those without. Exponentiated coefficients (exp[b]) were estimated from negative binomial regression, and as such, represent the factor change in the outcome variable in participants with a vascular risk factor relative to those without. Propensity scores with inverse probability treatment weighting were used to address potential confounders by balancing observed covariates including age, sex, years of education, marital status, and ethnocultural origins across exposure groups. These propensity score weights were used to adjust each regression model accordingly. All *P*-values of interest were adjusted using the Benjamini-Hochberg procedure based on false discovery rate (FDR) to generate FDR-corrected *q*-values Abbreviations: SCD, subjective cognitive decline; BMI, body mass index; ECog-II, Everyday Cognition II scale.

Having any number of VRFs was associated with greater severity of SCD symptoms, as measured by the ECog-II total score, in a dose-dependent manner (Table 2). Furthermore, all five individual VRFs were associated with greater severity of SCD symptoms. Diabetes exhibited the greatest effect: persons with diabetes had an ECog-II total score that was 1.40 times (95% CI [1.30–1.51], *q* < 0.001) greater than persons without diabetes, equivalent to approximately a 5-point rise in ECog-II total score from 11 to 16. Similarly, active smokers reported ECog-II total scores that were 1.31 (95% CI [1.22–1.41], *q* < 0.001) times higher than persons who had never smoked, although notably past smokers did not differ from non-smokers (exp[b] = 1.00, 95% CI [0.93–1.08], *q* = 0.93). The remaining VRFs of overweight, obese, hypertension, and high cholesterol were associated with 1.10–1.25 times higher ECog-II scores, as shown in Table 2. VRF associations with individual ECog-II domains are shown in Supplementary Fig. 1.

In contrast to SCD status, where only three or more VRFs were associated with greater risk, any number of VRFs were associated with greater odds of MBI status in a dose-dependent manner (Table 3). Likewise, all five individual VRFs were associated with greater odds of MBI + status (Table 3). Active smoking and obesity were most strongly linked to MBI + status: active smokers had more than twice the odds of MBI + status than non-smokers (OR = 2.67, 95% CI [2.25–3.18], *q* < 0.001), as did persons classified as obese relative to persons with normal BMI (OR = 2.29, 95% CI [1.91–2.75], *q* < 0.001). Like with SCD, VRF associations with MBI were generally stronger in the middle-aged group compared to the older adult group, particularly for overweight BMI, hypertension, and high cholesterol (Supplementary Table 2). Interestingly, diabetes and active smoking showed the opposite pattern with greater effects in older adults than in middle-aged adults. VRF associations with individual MBI-C domains are shown in Supplementary Fig. 2.

**Table 3:** Vascular risk factor associations with mild behavioural impairment

Outcome exposure	OR	95% CI	*p/q*
**MBI status**			
Cumulative VRF burden			
Low (1)	1.82	1.51–2.19	<0.001/<0.001
Moderate (2)	2.31	1.93––2.78	<0.001/<0.001
High (3+)	2.70	2.25–3.24	<0.001/<0.001
BMI (ref. normal BMI)			
Overweight	1.39	1.15–1.68	<0.001/0.001
Obese	2.29	1.91–2.75	<0.001/<0.001
Hypertension	1.58	1.33–1.89	<0.001/<0.001
High cholesterol	1.38	1.15–1.64	<0.001/<0.001
Diabetes	1.82	1.53–2.16	<0.001/<0.001
Smoking (ref. Never)			
Past smoker	1.38	1.15–1.65	<0.001/<0.001
Active smoker	2.67	2.25–3.18	<0.001/<0.001

	exp(b)	95% CI	*p/q*
**MBI-C total score**			
Cumulative VRF burden			
Low (1)	1.18	1.06–1.31	0.002/0.002
Moderate (2)	1.78	1.60–1.97	<0.001/<0.001
High (3+)	1.81	1.63–2.01	<0.001/<0.001
BMI (ref. normal BMI)			
Overweight	1.15	1.04–1.28	0.007/0.009
Obese	1.60	1.44–1.77	<0.001/<0.001
Hypertension	1.38	1.24–1.54	<0.001/<0.001
High cholesterol	1.22	1.10–1.35	<0.001/<0.001
Diabetes	1.36	1.24–1.50	<0.001/<0.001
Smoking (ref. Never)			
Past smoker	1.19	1.08–1.32	<0.001/<0.001
Active smoker	1.96	1.77–2.16	<0.001/<0.001

Odds ratios (ORs) were estimated from logistic regression; they indicate the factor change in odds of having MBI + between participants with a vascular risk factor relative to those without. Exponentiated coefficients (exp[b]) were estimated from negative binomial regression, and as such, represent the factor change in the outcome variable in participants with a vascular risk factor relative to those without. Propensity scores with inverse probability treatment weighting were used to address potential confounders by balancing observed covariates including age, sex, years of education, marital status, and ethnocultural origins across exposure groups. These propensity score weights were used to adjust each regression model accordingly. All *P*-values of interest were adjusted using the Benjamini-Hochberg procedure based on false discovery rate (FDR) to generate FDR-corrected *q*-values Abbreviations: MBI; mild behavioural impairment; BMI, body mass index; MBI-C, Mild Behavioural Impairment Checklist.

In line with VRF associations with MBI + status, both the cumulative number of VRFs and each of the five VRFs individually were all linked to higher MBI symptom severity, with active smoking and obesity showing the largest effects (Table 3). MBI symptoms were nearly doubled in active smokers compared to non-smokers (exp[b] = 1.96, 95% CI [1.77–2.16], *q* < 0.001), equivalent to approximately a 5-point rise in MBI-C scores from 5 to 10. Persons classified with obese also reported MBI symptoms that were 1.60 times (95% CI [1.44–1.77], *q* < 0.001) greater than those classified as having normal BMI. Similar associations were observed for hypertension, diabetes, past smoking, and having a BMI classification of overweight, as shown in Table 3.

**Figure 2:**
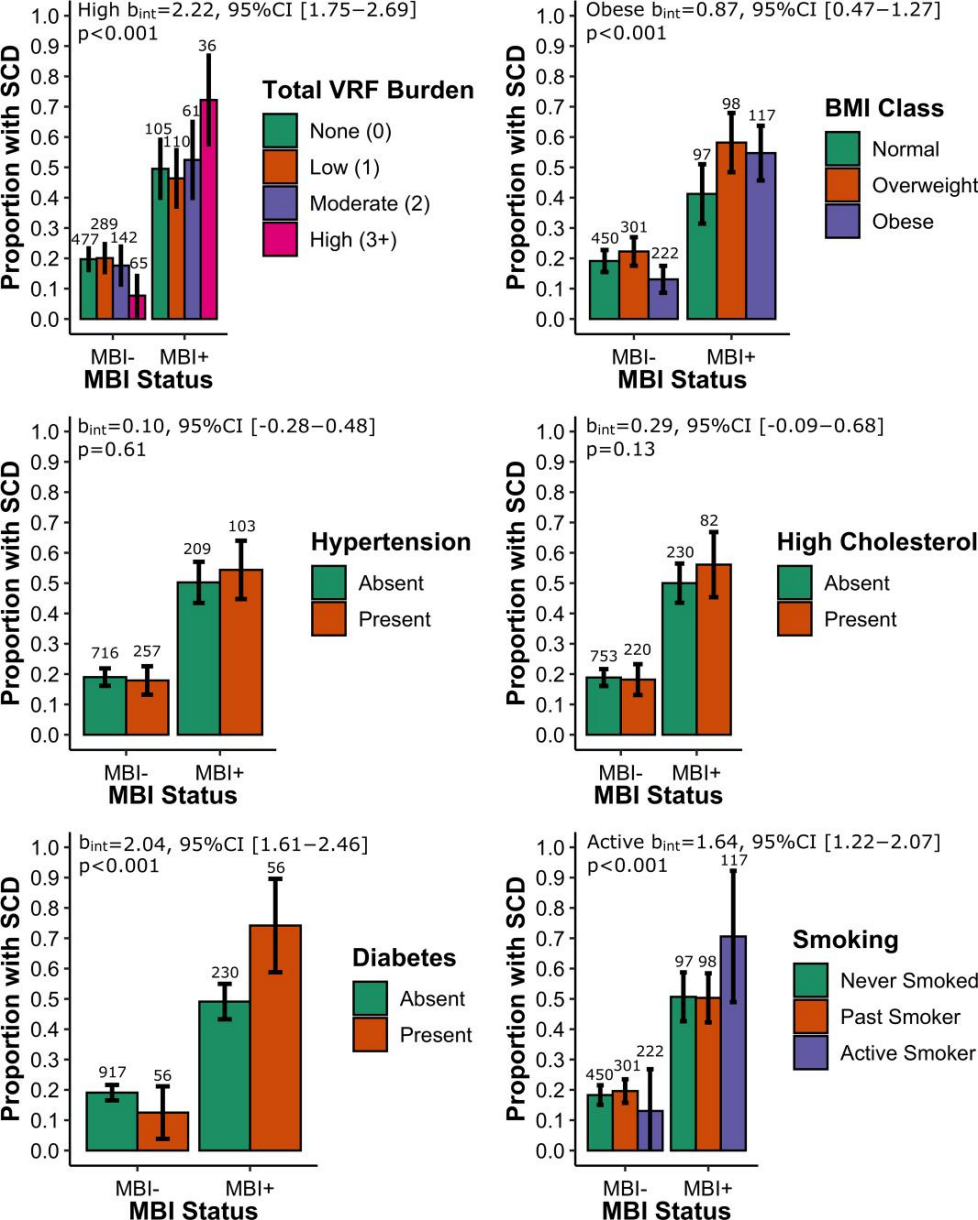
**Prevalence of SCD across VRF exposure groups among older persons with or without MBI.** Numbers above each bar indicate sample size for the group. Statistical outputs correspond to the coefficients of the VRF*MBI status interaction in a logistic regression model with SCD status as the outcome variable. Propensity scores with inverse probability treatment weighting were used to address potential confounders by balancing observed covariates including age, sex, years of education, marital status, and ethnocultural origins across exposure groups. Abbreviations: VRF, vascular risk factor; SCD, subjective cognitive decline; MBI, mild behavioural impairment; BMI, body mass index.

The secondary analysis revealed effect modification by MBI status for some of the VRFs in association with SCD. VRF variables were more strongly associated with SCD among MBI + individuals for the three or more VRFs group (MBI + OR = 2.21, 95% CI [1.44–3.39], *P* < 0.001 versus MBI − OR = 0.24, 95% CI [0.15–0.37], *P* < 0.001), obese BMI (MBI + OR = 1.63, 95% CI [1.11–2.38], *P* = 0.008 versus MBI − OR = 0.68, 95% CI [0.50–0.91], *P* = 0.007), diabetes (MBI + OR = 2.83, 95% CI [2.08–3.84], *P* < 0.001 versus MBI − OR = 0.37, 95% CI [0.27–0.49], *P* < 0.001), and active smoking (MBI + OR = 2.14, 95% CI [1.49–3.06], *P* < 0.001 versus MBI − OR = 0.41, 95% CI [0.29–0.59], *P* < 0.001) [Fig. 2]. Effect modification was not seen for less than three VRFs (low p_int_ = 0.32, moderate p_int_ = 0.29), overweight BMI (p_int_ = 0.08), hypertension (p_int_ = 0.61), high cholesterol (p_int_ = 0.13), and past smoking (p_int_ = 0.39).

## DISCUSSION

In a sample of community-dwelling older persons without MCI or dementia, having three or more VRFs, or individual VRFs of diabetes, active smoking, overweight, and obesity were linked to higher odds of having SCD. Having any number of VRFs, or individual VRFs of hypertension, high cholesterol, and past smoking were also associated with higher odds of having MBI. All VRFs were associated with more severe cognitive and behavioural symptoms in a dose-dependent manner, suggesting that greater management of VRFs may be beneficial for cognitive and behavioural health at older ages. Interestingly, associations of many VRFs with SCD were stronger in MBI + persons.

Our study is consistent with findings demonstrating relationships between VRFs and cognition. Poorer cardiovascular health score, comprising both the presence of VRFs and lifestyle factors, has been associated with higher dementia risk.^41^ Individual classification of higher BMI,^52^ diagnoses of hypertension,^53^ diabetes,^54-57^ and active smoking^58-61^ in mid- to late-life have all been independently associated with cognitive impairment, often even in advance of dementia. Furthermore, although hypertension and high cholesterol were not associated with higher odds of SCD in the present study, they were associated with more severe symptoms of SCD, consistent with existing literature.^62-64^ Several mechanisms explain how the presence of VRFs may contribute to downstream changes in cognition. For instance, hypertension is a strong and common risk factor for cerebral large and small vessel disease, and through subsequent vascular lesions, may give rise to cognitive impairment.^34,65,66^ Indeed, several forms of cerebral small vessel disease have been linked to cognitive impairment, even after accounting for other age-related neuropathologies, including Alzheimer’s disease.^67^ Vascular pathology may also interact with Alzheimer’s disease pathology to promote cognitive decline.^68^ Together, these findings indicate that vascular pathology, promoted by VRFs, may manifest as SCD at early stages of disease.

Later-life emergent and persistent changes in behaviour and personality (i.e. MBI) are increasingly recognized as primary sequelae of underlying neurodegenerative disease as opposed to simply secondary consequences of cognitive impairment.^12,20^ Yet, research surrounding MBI has largely focused on its function as a marker for Alzheimer’s disease.^69^ A growing body of evidence suggests a strong link between MBI and Alzheimer’s disease pathology, including greater amyloid-β, phosphorylated tau, and neurofilament light pathological burden,^20,24,70-74^ thus, enriching samples for preclinical or prodromal Alzheimer’s disease.^20^ However, just as cognitive impairment may arise from a variety of overlapping neurodegenerative and vascular aetiologies, so may MBI; studies of the latter have only recently begun to emerge. In two Singaporean studies examining hypertension, diabetes, and hyperlipidaemia, only diabetes was associated with MBI.^27,75^ Similarly, an Indian study identified a higher prevalence of diabetes among patients with MBI compared to those without.^76^ These associations were generally observed within a cohort of memory clinic patients, often with MCI. Our study provides evidence from a larger sample of community-dwelling older persons without objective cognitive impairment that BMI, hypertension, high cholesterol, diabetes, and smoking are all associated, to various degrees, with measurable changes in behaviour. Future research involving longitudinal data will be required to interrogate these relationships further and to elucidate underlying biological mechanisms.

The value of behavioural assessments in understanding the consequences of vascular pathology in the brain was highlighted not only by our finding that VRFs are associated with MBI, but also that VRF associations with cognition were generally stronger in older persons with MBI. In persons without MBI, VRF associations with cognition were weaker or not observed entirely. We propose two explanations for this finding. First, symptoms of MBI may directly influence VRF management at an individual level. Decreased motivation, for example, may lead to poorer adherence to VRF medications and lower participation in lifestyle activities, such as exercise, that are known to mitigate VRFs. As such, individuals with MBI may have more severe VRFs, resulting in more severe vascular pathology in the brain and greater cognitive impairment. Second, participants with MBI may represent a sub-cohort that is more likely to exhibit clinical symptoms in response to vascular pathology. This may be because older persons with MBI are more likely to also have preclinical Alzheimer’s disease, which when occurring in the presence of vascular pathology is known to lead to earlier symptom onset,^77^ or because they have a lower degree of resilience to early stages of brain pathology. Evidently, the mechanisms underlying this phenomenon remain poorly understood and replication in other cohorts is necessary. Nonetheless, future research on the effects of vascular pathology on cognition may benefit from consideration of behaviour. Clinically, these findings suggest that older adults with a high burden of VRF should be assessed for both cognitive changes and behavioural changes, both of which also serve as markers of elevated disease risk. Indeed, multidomain lifestyle interventions may be most effective in older adults at greater risk of dementia, as demonstrated by large clinical trials.^43^

The strength and direction of VRF associations with dementia are known to vary across the life course.^78-81^ Our study, which was conducted in a pooled sample of participants in midlife or later life, generally found that the presence of VRFs was associated with higher odds and severity of SCD and MBI symptoms. Subgroup analyses revealed that hypertension, overweight/obese BMI, and high cholesterol associations with SCD and MBI were stronger in middle-aged adults and attenuated in older adults, consistent with the literature.^57,82^ These patterns may be explained by a dynamic trajectory of VRFs over the disease course. Higher blood pressure, weight, and cholesterol levels may contribute to greater cerebrovascular pathological load in midlife, thereby impairing brain function and resulting in SCD and MBI. At later stages of disease, impaired brain function may be accompanied by sharply declining blood pressure, weight, and cholesterol levels, as has been observed at time points closely preceding a dementia diagnosis.^82^ Interestingly, certain VRFs such as diabetes and active smoking were more strongly associated with MBI in older adults than in middle-aged adults, suggesting that the moderating effect of age may work in the opposite direction for different VRFs. These findings warrant future studies with longitudinal designs to better understand how the trajectory of VRFs may be associated with SCD and MBI over time, and across different age groups.

One of the primary strengths of our study lies in the inclusion of a large premorbid cohort, allowing for robust examination of potentially modifiable associations pertaining to SCD and MBI. However, it is essential to acknowledge certain limitations. For example, while the fully online design of the study allows for greater recruitment, measures are mostly restricted to self-reports that cannot be fully validated using other sources or clinical assessments, including the clinical diagnoses of VRFs and cognitive statuses in this study. Replications in other cohorts are needed. The predominance of female volunteers, constituting 81% of the current sample, introduces a sex bias and stands as a potential limitation to the generalizability of our findings. Relatedly, the ethnocultural distribution of our cohort, comprising mostly of persons with European or North American origins, underscores the necessity for further investigations across more diverse ethnocultural samples. The cross-sectional design of our study precludes conclusions about causal pathways and mechanisms underlying the observed associations. Importantly, we were unable to thoroughly assess the impact of VRF medications on the observed associations as the vast majority of participants with VRFs reported taking medications for their VRFs. Along with medications for cognitive and behavioural symptoms, future studies should determine whether medications or lifestyle interventions can reliably impact the observed association between VRFs, cognition, and behaviour. Such studies may reveal therapeutic interventions for SCD and MBI symptoms, the latter of which has notably begun to garner attention in clinical trials.^33^

## Conclusion

Vascular pathologies are increasingly recognized as major contributors to brain health in older persons. Furthermore, unlike other neurodegenerative diseases, vascular pathologies may be reduced through stringent management of VRFs. We demonstrate that the presence of VRFs, including elevated BMI, hypertension, high cholesterol, diabetes, and active smoking are associated with SCD and MBI in community-dwelling older persons without MCI or dementia. Hence, prudent consideration should be given to the early mitigation of VRFs to potentially attenuate the likelihood of early cognitive and behavioural symptoms and future dementia incidence. Furthermore, more research is needed to better understand vascular links to behaviour and the role of behaviour in investigating vascular associations with cognition.

## Supplementary Material

fcaf163_Supplementary_Data

## Data Availability

Due to the confidential nature of participant demographic health data, CAN-PROTECT study data is not publicly available on a data repository. However, individual access to the data may be provided upon reasonable request to the corresponding author. No specialized in-house scripts or programmes were used for this study.
