# Supplementary material for: Vascular risk factor associations with subjective cognitive decline and mild behavioural impairment
Source: Brain Commun. 2025 Apr 28;7(3):fcaf163. doi: 10.1093/braincomms/fcaf163 (PMC12077299; doi:10.1093/braincomms/fcaf163)
Supplement: fcaf163_Supplementary_Data [file fcaf163_supplementary_data.docx]

**SUPPLEMENTARY MATERIALS**

| **Supplementary Table 1. ECog-II and MBI-C domain characteristics stratified by cumulative vascular risk factor burden** | | | | | | |
| --- | --- | --- | --- | --- | --- | --- |
| **Variable** | **Total** | **None (0)** | **Low (1)** | **Moderate (2)** | **High (3+)** | **p** |
| n | 1285 | 582 | 399 | 203 | 101 |  |
| SCD status | 343 (26.7) | 146 (25.1) | 109 (27.3) | 57 (28.1) | 31 (30.7) | 0.60 |
| ECog-II |  |  |  |  |  |  |
| Total | 11.6 (11.2),  0-99 | 10.5 (9.9),  0-74 | 11.8 (11.4),  0-88 | 13 (13), 0-99 | 14.3 (12.9), 0-65 | 0.002 |
| Memory | 4.6 (3.7),  0-25 | 4.2 (3.5),  0-24 | 4.8 (3.9),  0-20 | 5.1 (4.0),  0-25 | 5.2 (4.0),  0-21 | 0.003 |
| Language | 3.2 (3.4),  0-24 | 2.9 (3.2),  0-21 | 3.3 (3.6),  0-24 | 3.3 (3.5),  0-21 | 3.7 (3.5),  0-15 | 0.06 |
| Visuospatial | 0.8 (1.5),  0-18 | 0.7 (1.2),  0-10 | 0.9 (1.6),  0-14 | 0.8 (1.7),  0-18 | 0.9 (1.5),  0-10 | 0.27 |
| Executive Function | 3.0 (4.4),  0-39 | 2.7 (3.6),  0-25 | 2.8 (4.1),  0-35 | 3.7 (5.6),  0-39 | 4.5 (5.9),  0-31 | <0.001 |
| MBI presence (MBI-C cutoff) |  |  |  |  |  |  |
| Global (≥8) | 312 (24.3) | 105 (18) | 110 (27.6) | 61 (30) | 36 (35.6) | <0.001 |
| Decreased  motivation (≥1) | 306 (23.8) | 103 (17.7) | 107 (26.8) | 61 (30) | 35 (34.7) | <0.001 |
| Affective  dysregulation (≥1) | 304 (23.7) | 103 (17.7) | 107 (26.8) | 59 (29.1) | 35 (34.7) | <0.001 |
| Impulse dyscontrol  (≥1) | 293 (22.8) | 97 (16.7) | 101 (25.3) | 60 (29.6) | 35 (34.7) | <0.001 |
| Social  inappropriateness  (≥1) | 116 (9.0) | 33 (5.7) | 34 (8.5) | 31 (15.3) | 18 (17.8) | <0.001 |
| Psychosis (≥1) | 126 (9.8) | 46 (7.9) | 34 (8.5) | 25 (12.3) | 21 (20.8) | <0.001 |
| MBI-C Severity |  |  |  |  |  |  |
| Global | 5.2 (7.2),  0-65 | 4.3 (6.2),  0-44 | 5 (5.9),  0-39 | 6.9 (10), 0-65 | 7.5 (9.3), 0-41 | <0.001 |
| Decreased  motivation | 5.2 (7.2),  0-65.0 | 4.3 (6.2),  0-44.0 | 5.0 (5.9),  0-39.0 | 6.9 (10), 0-65.0 | 7.5 (9.3), 0-41.0 | <0.001 |
| Affective  dysregulation | 1.6 (2.5),  0-18.0 | 1.4 (2.2),  0-16.0 | 1.7 (2.4),  0-12.0 | 1.9 (3.0), 0-18.0 | 2.4 (3.2), 0-14.0 | <0.001 |
| Impulse dyscontrol | 1.7 (2.5),  0-16.0 | 1.5 (2.4),  0-16.0 | 1.7 (2.3),  0-15.0 | 2.1 (2.9), 0-15.0 | 2.1 (3.2), 0-16.0 | 0.01 |
| Social  inappropriateness | 1.5 (2.4),  0-20 | 1.2 (1.9),  0-17.0 | 1.3 (1.7),  0-11.0 | 2.3 (3.6), 0-20 | 2.2 (2.9), 0-12.0 | <0.001 |
| Psychosis | 0.2 (0.7),  0-9.0 | 0.2 (0.6),  0-7.0 | 0.2 (0.6),  0-5.0 | 0.4 (1.1), 0-9.0 | 0.4 (0.8), 0-5.0 | <0.001 |
| *Note*. All values have been rounded to one decimal place, except for p-values which have been rounded to two or three decimal places, as appropriate. Continuous variables are shown in mean (standard deviation), range. Categorical variables are shown in n (%). Comparisons between groups were tested using ANOVA for continuous variables and chi-square tests for categorical variables, as appropriate. Abbreviations: SCD, subjective cognitive decline; ECog-II; Everyday Cognition II scale; MBI, mild behavioral impairment; MBI-C, Mild Behavioral Impairment Checklist. | | | | | | |

| **Supplementary Table 2. Vascular risk factor associations with subjective cognition decline and mild behavioral impairment in middle-aged and older adults** | | | | | | |
| --- | --- | --- | --- | --- | --- | --- |
| **Outcome** | **Middle-Aged (<65 years; n=657)** | | | **Older Adult (≥65 years; n=628)** | | |
| **Exposure** | **OR** | **95% CI** | **p** | **OR** | **95% CI** | **p** |
| **SCD status** |  |  |  |  |  |  |
| Cumulative VRF burden |  |  |  |  |  |  |
| Low (1) | 0.89 | 0.70–1.14 | 0.37 | 1.50 | 1.16–1.94 | 0.002 |
| Moderate (2) | 1.19 | 0.94–1.51 | 0.15 | 1.19 | 0.91–1.55 | 0.20 |
| High (3+) | 1.25 | 0.98–1.58 | 0.07 | 1.07 | 0.82–1.41 | 0.60 |
| BMI (ref. normal BMI) |  |  |  |  |  |  |
| Overweight | 2.08 | 1.64–2.65 | <0.001 | 1.09 | 0.84–1.41 | 0.52 |
| Obese | 1.26 | 0.98–1.62 | 0.07 | 1.24 | 0.96–1.60 | 0.09 |
| Hypertension | 1.23 | 0.96–1.56 | 0.10 | 1.01 | 0.78–1.30 | 0.95 |
| High cholesterol | 1.14 | 0.90–1.45 | 0.27 | 1.11 | 0.86–1.43 | 0.44 |
| Diabetes | 1.08 | 0.85–1.37 | 0.54 | 1.23 | 0.95–1.58 | 0.11 |
| Smoking (ref. Never) |  |  |  |  |  |  |
| Past smoker | 1.20 | 0.94–1.52 | 0.14 | 1.04 | 0.81–1.34 | 0.75 |
| Active smoker | 1.36 | 1.08–1.73 | 0.01 | 1.26 | 0.98–1.63 | 0.07 |
|  |  |  |  |  |  |  |
|  | **OR** | **95% CI** | **p** | **OR** | **95% CI** | **p** |
| **MBI status** |  |  |  |  |  |  |
| Cumulative VRF burden |  |  |  |  |  |  |
| Low (1) | 1.60 | 1.25–2.05 | <0.001 | 2.00 | 1.49–2.68 | <0.001 |
| Moderate (2) | 2.75 | 2.16–3.49 | <0.001 | 1.73 | 1.29–2.34 | <0.001 |
| High (3+) | 2.73 | 2.15–3.47 | <0.001 | 2.45 | 1.84–3.28 | <0.001 |
| BMI (ref. normal BMI) |  |  |  |  |  |  |
| Overweight | 1.42 | 1.11–1.83 | 0.006 | 1.20 | 0.89–1.62 | 0.23 |
| Obese | 2.27 | 1.78–2.89 | <0.001 | 2.31 | 1.75–3.06 | <0.001 |
| Hypertension | 1.92 | 1.52–2.43 | <0.001 | 1.23 | 0.93–1.62 | 0.14 |
| High cholesterol | 1.78 | 1.41–2.25 | <0.001 | 0.99 | 0.75–1.31 | 0.97 |
| Diabetes | 1.41 | 1.12–1.78 | 0.004 | 2.03 | 1.56–2.64 | <0.001 |
| Smoking (ref. Never) |  |  |  |  |  |  |
| Past smoker | 1.66 | 1.31–2.11 | <0.001 | 1.12 | 0.84–1.48 | 0.45 |
| Active smoker | 1.82 | 1.44–2.31 | <0.001 | 2.81 | 2.16–3.65 | <0.001 |
| Odds ratios (ORs) were estimated from logistic regression; they indicate the factor change in odds of having SCD between participants with a vascular risk factor relative to those without. Exponentiated coefficients (exp[b]) were estimated from negative binomial regression, and as such, represent the factor change in the outcome variable in participants with a vascular risk factor relative to those without. Propensity scores with inverse probability treatment weighting were used to address potential confounders by balancing observed covariates including age, sex, years of education, marital status, and ethnocultural origins across exposure groups. These propensity score weights were used to adjust each regression model accordingly. All p-values of interest were adjusted using the Benjamini-Hochberg procedure based on false discovery rate (FDR) to generate FDR-corrected q-values Abbreviations: SCD, subjective cognitive decline; MBI, mild behavioral impairment; VRF, vascular risk factor; BMI, body mass index. | | | | | | |


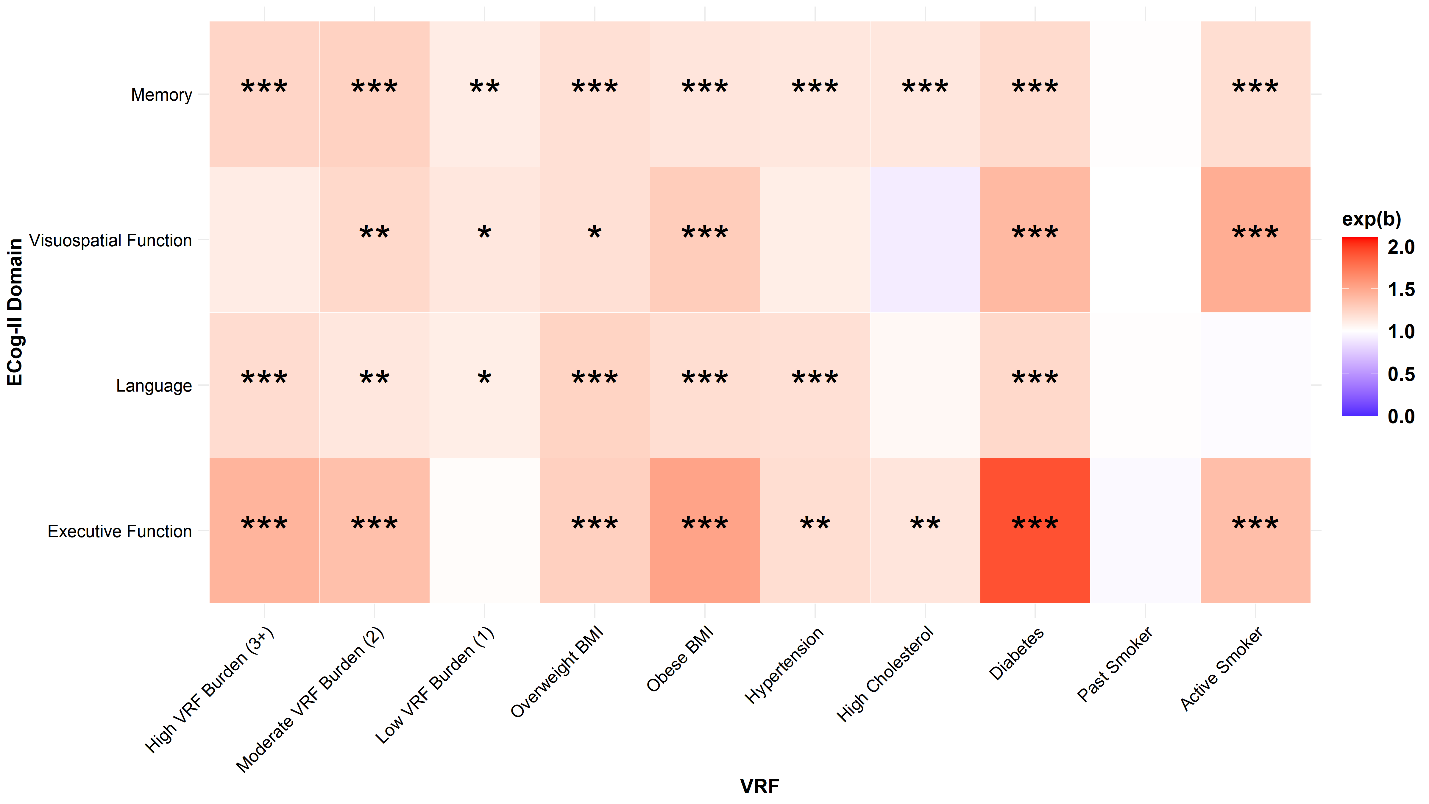


**Supplementary Figure 1. Domain-specific associations between vascular risk factors and ECog-II domains.** Exponentiated coefficients (exp[b]) were estimated from negative binomial regression, and as such, represent the factor change in the outcome variable in participants with a vascular risk factor relative to those without. Propensity scores with inverse probability treatment weighting were used to address potential confounders by balancing observed covariates including age, sex, years of education, marital status, and ethnocultural origins across exposure groups. These propensity score weights were used to adjust each regression model accordingly. Abbreviations: VRF, vascular risk factor; ECog-II, Everyday Cognition Scale II; BMI, body mass index.

**
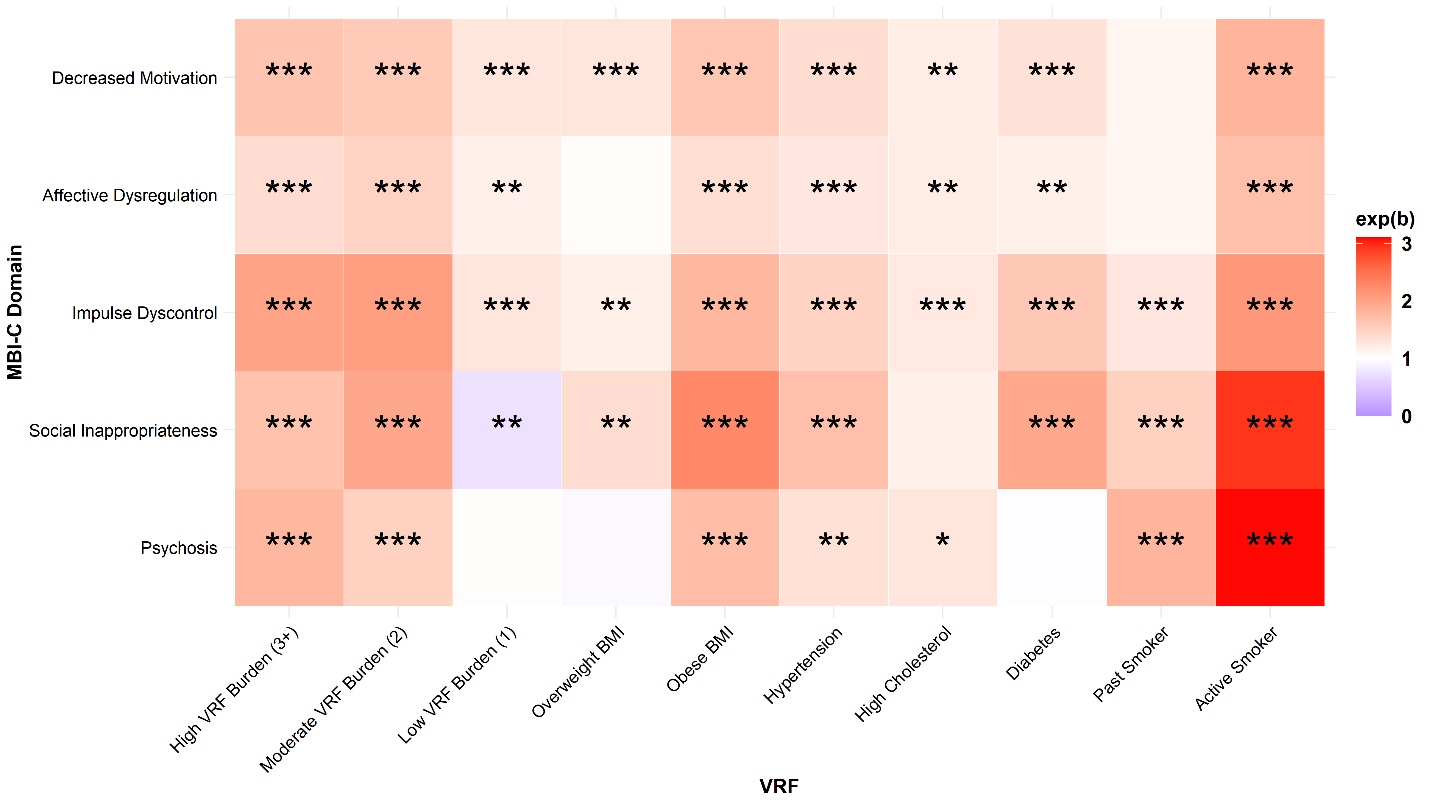
**

**Supplementary Figure 2. Domain-specific associations between vascular risk factors and MBI-C domains.** Exponentiated coefficients (exp[b]) were estimated from negative binomial regression, and as such, represent the factor change in the outcome variable in participants with a vascular risk factor relative to those without. Propensity scores with inverse probability treatment weighting were used to address potential confounders by balancing observed covariates including age, sex, years of education, marital status, and ethnocultural origins across exposure groups. Abbreviations: VRF, vascular risk factor; MBI-C, Mild Behavioral Impairment Checklist; BMI, body mass index.
